# Supplementary figures and images for: Comparison of In-Frame Deletion, Homology-Directed Repair, and Prime Editing-Based Correction of Duchenne Muscular Dystrophy Mutations
Source: Biomolecules. 2023 May 22;13(5):870. doi: 10.3390/biom13050870 (PMC10216110; doi:10.3390/biom13050870)

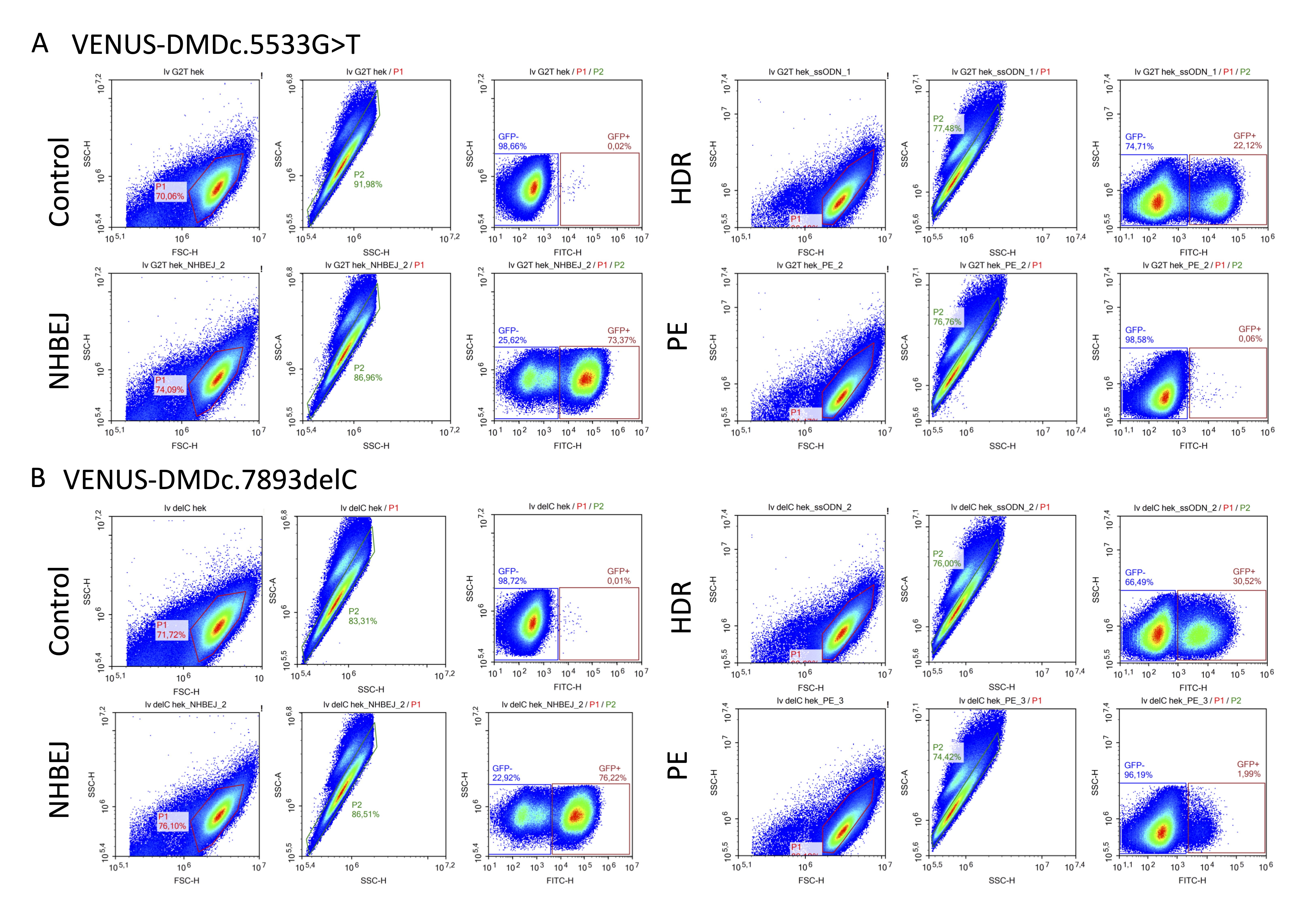

Supplement: Supplementary file 1 [file biomolecules-13-00870-s001.zip › VENUS Figure S1 FACS-based quantification of EGFP+ cells.jpg]
